# Study of the influence of tributyrin-supplemented diets on the gut bacterial communities of rainbow trout (*Oncorhynchus mykiss*)

**DOI:** 10.1038/s41598-024-55660-y

**Published:** 2024-03-07

**Authors:** A. Louvado, F. J. R. C. Coelho, M. Palma, L. J. Magnoni, F. Silva-Brito, R. O. A. Ozório, D. F. R. Cleary, I. Viegas, N. C. M. Gomes

**Affiliations:** 1 Centre for Environmental and Marine Studies (CESAM) & Department of Biology, University of Aveiro, 3810-193 Aveiro, Portugal; 2 Centre for Functional Ecology, Associate Laboratory TERRA, Department of Life Sciences, University of Coimbra, 3000-456 Coimbra, Portugal; 3 Centro Interdisciplinar de Investigação Marinha e Ambiental, Universidade do Porto, Matosinhos, Portugal; 4 The New Zealand Institute for Plant and Food Research Limited, Nelson, New Zealand

**Keywords:** Applied microbiology, Marine biology, Microbial ecology, Metabolomics, Microbial ecology

## Abstract

Dietary supplementation with triglyceride tributyrin (TBT), a butyrate precursor, has been associated with beneficial effects on fish health and improvements in the ability of carnivorous fish to tolerate higher levels of plant-based protein. In this study, we aimed to investigate the effects of a plant-based diet supplemented with TBT on the structural diversity and putative function of the digesta-associated bacterial communities of rainbow trout (*Oncorhynchus mykiss*). In addition to this, we also assessed the response of fish gut digestive enzyme activities and chyme metabolic profile in response to TBT supplementation. Our results indicated that TBT had no significant effects on the overall fish gut bacterial communities, digestive enzyme activities or metabolic profile when compared with non-supplemented controls. However, a more in-depth analysis into the most abundant taxa showed that diets at the highest TBT concentrations (0.2% and 0.4%) selectively inhibited members of the Enterobacterales order and reduced the relative abundance of a bacterial population related to *Klebsiella pneumoniae*, a potential fish pathogen. Furthermore, the predicted functional analysis of the bacterial communities indicated that increased levels of TBT were associated with depleted KEGG pathways related to pathogenesis. The specific effects of TBT on gut bacterial communities observed here are intriguing and encourage further studies to investigate the potential of this triglyceride to promote pathogen suppression in the fish gut environment, namely in the context of aquaculture.

## Introduction

The farming of carnivorous fish depends to a considerable extent on fish-derived fishmeal as the primary source of protein^1^. The decline in natural marine resources, however, has stimulated a search for alternative raw materials that can complement proteins of animal origin in fish feeds^2^. The use of proteins of plant origin is the major contributor for aquaculture feeds^1^. However, the inclusion of increasing amounts of plant-based protein in aquafeeds are often associated with lower growth rates, changes to fish metabolism, increased morbidity, deficiencies in essential amino acids and changes to fish-associated microbial communities^3^. Research has shown that carnivorous fish diets supplemented with butyrate, a short chain fatty acid (SCFA), help fish to withstand substantial levels of plant-based protein, and, simultaneously, improve intestinal epithelial permeability and mucus production, reduce gut inflammation and stabilize gut microbiota^4–6^.

SCFAs are often found in the intestine as a result of the metabolic by-products of gut microbial conversion of dietary carbohydrates^7^. They have been the subject to much research in recent years, particularly in humans^7^. SCFAs have several beneficial impacts on the human metabolism such as glucose homeostasis, lipid metabolism, intestinal barrier integrity and host immunity response^8^. The main SCFA produced by gut bacteria are acetate, propionate and butyrate^9^. Butyrate has been shown to have a positive effect on growth performance, feed efficiency, antioxidant factors and immunological responses in several aquatic species^10^. Supplementation with butyrate and its derivate salts such as sodium butyrate have been shown to improve growth parameters, feed conversion ratios and intestinal villous height in pirarucu juveniles (*Arapaima gigas*)^11^. Butyrate also reduced aspartate aminotransferase activity and increased glycogen levels, helping the animals to better resist the stressful conditions found in an intensive aquaculture system^12^. Moreover, experimental trials have shown that a 0.5% (m/m) dose of 1-monobutyrin can effectively modulate the microbiome of the marine fish (*Sparus aurata*), with seabream fed SCFA having more beneficial lactic acid bacteria (i.e., *Lactobacillus* sp.), and less potential fish pathogens^13^. The use of butyrate in feeds, however, is challenging. Its strong odor and bitter taste make it less appealing to fish and it has a limited half-life^14^.

Currently, tributyrin [2,3-di(butanoyloxy)propyl butanoate; TBT], a non-volatile stable triglyceride is seen as an alternative to the direct use of butyrate^15^. This compound generates three molecules of butyrate and one of glycerol via intestinal lipolysis and its utilization has been associated with positive effects on the growth and intestinal digestive and barrier functions of fish^16–18^ and shrimp^19^. However, despite the potential role of TBT to improve fish health and the ability of carnivorous fish to tolerate higher levels of plant-based protein, until now only a few studies have investigated the effects of TBT on the stability of fish-associated microbial communities^17,18^.

In this study, for the first time, we investigated the response of the structural diversity and putative functions of digesta-associated intestinal microbiota of rainbow trout (*Oncorhynchus mykiss*) to TBT supplementation in a plant-based diet. In addition to this, we also assessed the response of fish gut digestive enzyme activities and metabolome profile of the digesta to TBT. Rainbow trout is one of the most important cultured freshwater fish species in the world, with an important economic role in European aquaculture^20^. Rainbow trout is the second most produced finfish in Europe and, in 2019 Europe was the world’s second largest producer of trout^20,21^. Studies aiming to develop and optimize plant-based feed in the aquaculture sector can promote more economically and environmentally sustainable aquaculture practices.

## Material and methods

### Experimental design

This study was conducted under the supervision of accredited experts in laboratory animal science by the Portuguese Veterinary Authority (1005/92, DGAV-Portugal, following FELASA category C recommendations), according to the guidelines on the protection of animals used for scientific purposes from the European directive 2010/63/UE. The fish trial took place at the aquaculture facilities of the Interdisciplinary Centre of Marine and Environmental Research (CIIMAR). The study was designed to comply with the ARRIVE (Animal Research: Reporting of In Vivo Experiments) guidelines for reporting of animal research. The experimental protocol was approved by the Animal Welfare and Ethics Body Committee of CIIMAR (ORBEA-CIIMAR 08-2017), with the final authorization from the General Directorate for Food and Veterinary Medicine (ID: 0421/000/000/2020).

Juvenile rainbow trout were produced by “Truticultura do Minho” (Paredes de Coura, Portugal) and transported to the quarantine facilities of BOGA, where they were kept for 15 days after arrival. Thereafter, four hundred and eighty fish were randomly distributed in 12 tanks of 210 L each and fed twice a day with a commercial diet (SPAROS Lda, Olhão, Portugal) at a maintenance ratio (2% body weight day^−1^) for 12 days before the start of the trial. Experimental tanks were connected in a recirculated water system, with monitored physicochemical conditions daily (temperature = 16 ± 1 °C; salinity = 0.30 ± 1‰; pH = 7 ± 1 and air saturation > 95%). Water ammonium and nitrite levels were maintained below 0.05 mg mL^−1^ and 0.5 mg mL^−1^, respectively, throughout the experiment and the photoperiod regime was a 12 h:12 h day:night cycle.

### Feeding trial

In the experimental feeding trial, each tank was randomly assigned to one of the dietary treatments as follows: basal control diet (Cont), diet supplemented with a tributyrin product (TBT, supplied by Lucta S.A., Barcelona, Spain) at 0.1% (m/m) (TBT1), 0.2% (TBT2), and 0.4% (TBT4) (triplicate tanks per diet). This product consisted of 98% purity tributyrin (55%) and silica carrier (45%). A detailed description of the formulation of each diet can be found in Supplementary Table S1. Despite using 60% plant-based meals, it is worth noting that this was not a fishmeal-free diet, and 10% fishmeal was included. This formulation was developed to be nutritionally challenging but with a realistic application in the commercial context. Fish were hand-fed twice a day to apparent satiety at 9:00 h and 16:00 h during 50 days. Rainbow trout had an initial body weight (IBW) of 18.99 ± 0.19 g (mean ± SE) and a final body weight (FBW) of 63.23 ± 1.02 g at the end of the trial. No significant differences were found in the IBW (*P* = 0.478).

### Sampling

Fish were sampled 24 h after last meal, anesthetized with MS-222 (0.1 g L^−1^) buffered with NaHCO_3_ (0.2 g L^−1^), and euthanized by cervical section. Six fish were euthanized per tank. From three of these fish (n = 9 per diet), intestinal and digesta samples were collected to assess digestive enzyme activities and metabolome, respectively. These samples were immediately frozen with liquid nitrogen and then stored at – 80 °C. The other three fish (n = 9 per diet) were used to obtain digesta samples for microbiome analysis. Digesta samples were obtained by intestinal stripping into a sterile tube. Digesta samples for microbiome analysis were combined with 1 mL of RNAlater^®^ (Qiagen, Hilden, Germany) and keep in ice during transport and DNA extraction was performed 2 h after sampling. No additional storage was required.

### Digestive enzyme activities

Activities were measured in samples of individual fish. The whole intestinal tract including pyloric ceca was homogenized with a Potter Elvehjem homogenizer (Thomas Scientific LLC, Swedesboro, NJ, USA) in buffer solution (1:5, w/v), made by 50 mM Tris–HCl and 200 mM NaCl at pH 8.0 (Sigma-Aldrich, St. Louis, MO, USA). Homogenates were centrifuged at 13,500×*g* at 4 °C for 30 min. After discarding the lipid layer, the supernatant was used to determine the enzymatic activities in a microplate reader (Synergy HT, Biotek SA, Winooski, VT, USA). Alpha-amylase (EC 3.2.1.1), lipase (EC 3.1.1.3), trypsin (EC 3.4.21.4), and chymotrypsin (EC 3.4.21.1) activities were measured as previously described^6,22^. Total protein concentration in the supernatants was quantified by the Folin phenol method^23^ and the value was used to calculate the specific enzyme activity. Protein concentration was determined by reference to a standard curve of bovine γ-globulin (Sigma-Aldrich). A one-way ANOVA analysis was performed to test the effect of the experimental diet treatments on digestive enzyme activities using SigmaPlot for Windows version 14.5 (Systat Software Inc., Palo Alto, CA, USA). Data were checked for normality and homogeneity of variance (Shapiro–Wilk and Brown-Forsythe tests, respectively). Significant differences were considered when *P* < 0.05.

### DNA extraction

Digesta was pelleted by centrifugation at 16,000×*g* during 1 min and the preservative removed. DNA extraction was performed using the Qiagen Mini Stool Kit (Qiagen, Hilden, Germany) following the manufacturer’s instructions with some modifications to the initial steps. A bead-beating step was introduced at the beginning of the extraction. Samples and buffer solution from the kit were transferred to bead-beating tubes containing lysing matrix E (MP Biomedicals, Santa Ana, CA, USA) and ground twice using the Fastprep-24 bead-beating grinder (MP Biomedicals) set at 6.5 m s^−1^ during 45 s, with a resting period of 60 s. DNA was stored at − 20 °C until further use.

### Illumina MiSeq sequencing

The hypervariable V4 region of the 16S rRNA gene was amplified by PCR using the primers 515F (GTGCCAGCMGCCGCGGTAA) and 806R (GGACTACHVGGGTWTCTAAT). Library preparation and sequencing were performed using a MiSeq sequencing platform at the Molecular Research LP (www.mrdnalab.com; Shallowater, TX, USA), following standard Illumina procedures (Illumina, San Diego, CA, USA). QIIME2 (version 2020.8) was used to transform the amplicon libraries to an amplicon sequence variant (ASV) abundance table^24^. Demultiplexing was preformed using the “demux” algorithm in QIIME2. The “dada2” algorithm of the DADA2 plugin^25^ in QIIME2 was used to filter low quality reads, merge forward and reverse reads into sequences, remove chimeras and group sequences into ASVs. In dada2, forward and reverse sequences were both truncated at 220 bp. Taxonomy was assigned to representative sequences of ASVs by the ‘feature-classifier’ algorithm in QIIME2 using a scikit-learn Naïve Bayes classifier based on the SILVA database of the 16S reference sequences at 99% similarity (version 138, released December 2019^26^), made available at docs.qiime2.org^27,28^. To simplify interpretation, a unique number was assigned to each ASV. Non-bacterial, mitochondrial and chloroplast sequences were removed. A negative control from the DNA extraction was also sequenced and all ASVs that occurred in the negative control were removed, with the exception of ASV2. This ASV had a much higher abundance in the majority of the fish samples when compared with the negative control and we assume that its presence in the negative control was most likely a result of “index hopping”^29^. A list of removed ASVs is available in Supplementary File S1.

### Data analysis

A table containing the ASV counts per sample was imported into R and used to compare community diversity, composition and assess the relative abundances of selected higher taxa. Richness, Shannon's H', Peilou’s J (evenness) and Fisher's alpha diversity were obtained using the rarefy() and diversity() functions from the vegan package^30^. We tested for significant differences in community diversity and the relative abundances of the most abundant taxonomic groups and ASVs with an analysis of deviance using the glm() function from the stats package^31^. Since there were a number of samples with zero counts, we set the family argument to ‘tweedie’ using the tweedie() function from the tweedie package with var.power = 1.5 and link.power = 0 (a compound Poisson–gamma distribution). Using the GLM model, we tested for significant variation among treatments using the anova() function from the vegan package with the F test selected. The emmeans() function in the emmeans library was used to perform post-hoc multiple pairwise comparisons of mean abundance among diets using the false discovery rate (fdr) method in the adjust argument. The ordinate() function from the phyloseq package was used for compositional analysis^32^. We created a phyloseq object using the phyloseq() function from the phyloseq package. The input for the function included the ASV table, taxonomic metadata for the ASV table and metadata for each sample. The ASV table was rarefied for the ordination analysis with the rarefy_even_depth() function from the phyloseq package. The ordinate() function was then used with the phyloseq object as input, the method argument set to 'PCoA' and the distance argument set to 'bray'. The plot_ordination() function from the phyloseq package with the argument set to 'biplot' was used to produce the ordination plot. Significant differences in composition among treatments were tested using a permutational multivariate analysis of variance (PERMANOVA) with the adonis() function in the vegan package, with permutations set at 999. The BLAST search tool was used to compare sequences from ASVs 1, 2, 3, and 4 with sequences in NCBI’s curated 16S ribosomal RNA sequences (Bacteria and Archaea type strains) database. The top 100 results are shown in Supplementary File S3.

The Tax4Fun2 package in R^33^ was used to predict the metagenomic content of each sample based on the three-tier KEGG (Kyoto Encyclopedia of Genes and Genomes) orthology^34^. First, the runRefBlast() function with the database mode was set to “Ref100NR” and the path_to_otus argument set to the representative sequences file generated using QIIME2. The makeFunctionalPrediction() function was then used with the path_to_otu_table argument set to the ASV table and the min_identity_to_reference argument set to 0.95. Default settings were used for the other arguments.

### Digesta NMR-metabolomics

Digesta samples from fish sampled 24 h after last meal (n = 36) were thawed and kept on ice throughout the process. Digesta was weighted and processed as previously described^35^ but with a waiting time of 30 min before centrifugation. The aqueous fraction of digesta-chyme (final supernatant) was recovered and 200 μL of each sample were lyophilized and stored at -20 °C until analysis by Nuclear Magnetic Resonance (NMR). Samples were prepared by adding 19 μL of phosphate buffer (1.572 M; pD 7.41; with 1.91 mM sodium formate and 5.01 mM 3-(Trimethylsilyl) propionic-2,2,3,3-d_4_ acid sodium salt (TSP) in 99.8% ^2^H_2_O) and 201 μL 99.8% ^2^H_2_O. Proton (^1^H) NMR spectroscopy was performed on a Varian VNMRS 600 MHz (Agilent, Santa Clara, CA, USA) spectrometer, equipped with a 3-mm ^1^H(X)-PFG inverse configuration probe. A CPMG pulse sequence was acquired for each sample (spectral width 7 kHz; acquisition time 3 s; saturation delay 2 s; relaxation delay 2 s; 64 scans; 512 ms echo-time; at 298 K). Spectra were processed and targeted analysis was manually performed as described previously^35^. The raw spectra obtained during the current study have been uploaded to the Zenodo repository (https://zenodo.org) with the reference doi: 10.5281/zenodo.6671278. Principal Components Analysis (PCA) was performed using MetaboAnalyst 5.0 software (https://www.metaboanalyst.ca).

## Results and discussion

**Figure 1:**
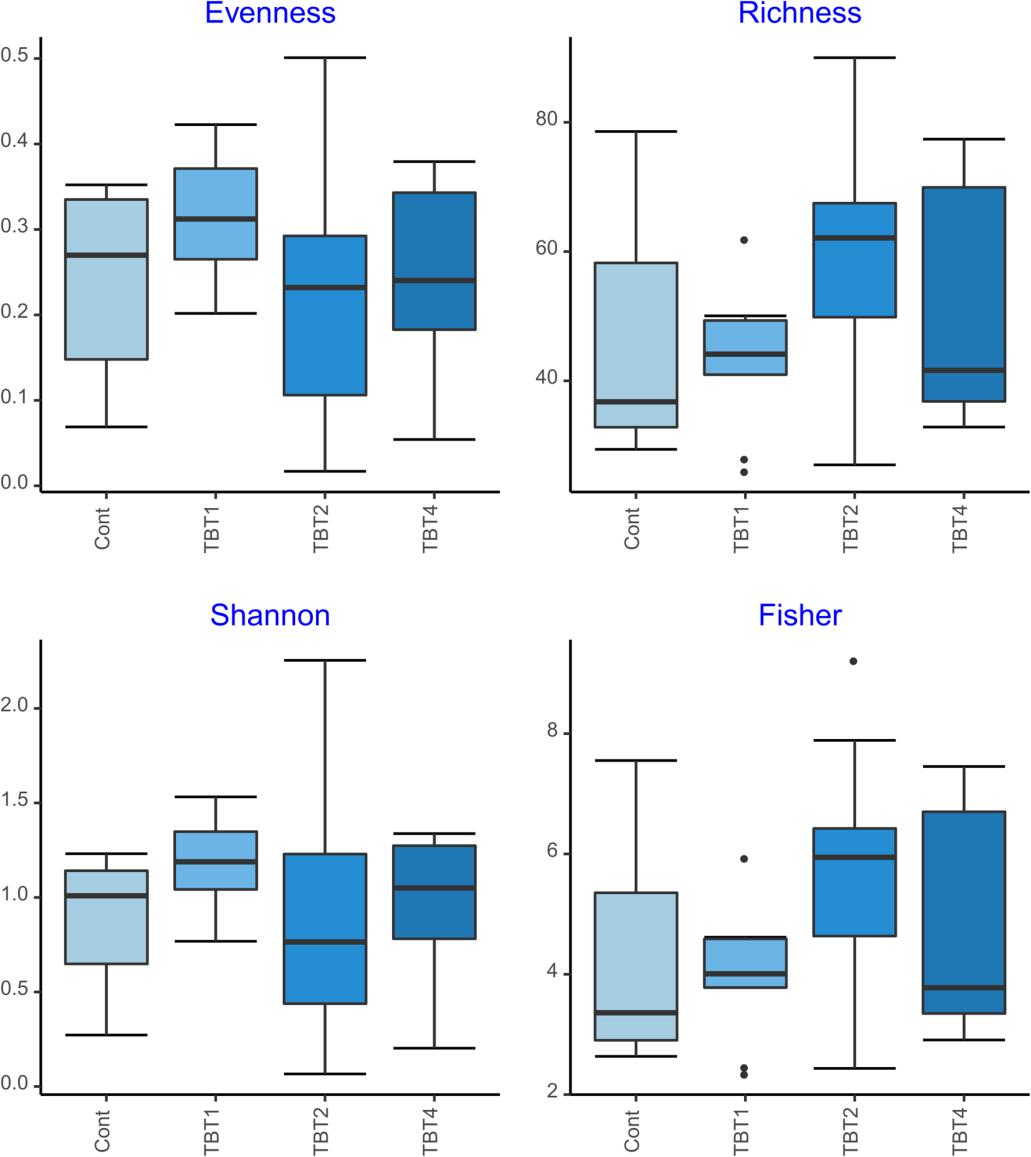
Diversity indices [Peilou’s J (Evenness), Richness, Shannon’s H’ (Shannon) and Fisher’s α (Fisher)] of digesta-bacterial communities of fish fed diets with increasing levels of tributyrin (TBT). Cont: basal diet only (no TBT added); TBT1: basal diet with 0.1% TBT (m/m); TBT2: basal diet with 0.2% TBT (m/m); TBT4: basal diet with 0.4% TBT (m/m). In the experimental diets, TBT was added to the basal diet at the expense of the excipient silica. Symbols beyond the ends of the whiskers are outliers.

**Figure 2:**
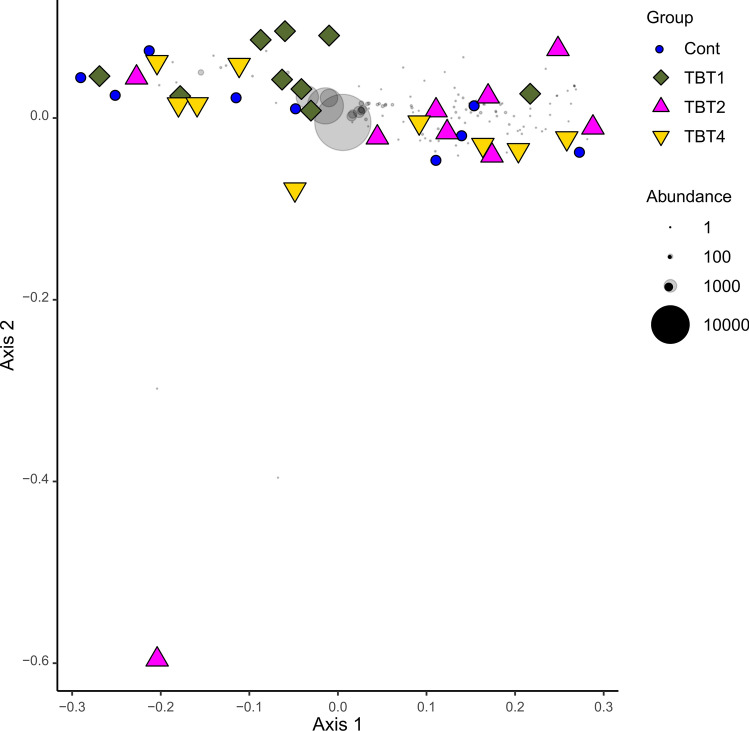
Ordination showing the first two axes of the principal coordinates analysis of ASV composition of digesta-bacterial communities of fish fed diets with increasing levels of tributyrin (TBT). Symbols are colour-coded and represent samples from different diets as shown in the legend on the right side of the figure. Grey symbols represent weighted averages scores for ASVs. The symbol size is proportional to group abundance (number of sequence reads). Cont: basal diet only (no TBT added); TBT1: basal diet with 0.1% TBT (m/m); TBT2: basal diet with 0.2% TBT (m/m); TBT4: basal diet with 0.4% TBT (m/m). In experimental diets, TBT was added to the basal diet at the expense of the excipient silica.

The bacterial community dataset consisted of 8,948,681 sequences grouped into 435 ASVs after quality control. No significant differences were found among treatments in evenness, richness, Shannon’s H’, and Fisher’s diversity indices (GLM-ANOVA *p* < 0.05; Fig. 1, Supplementary File S4). The ordination also did not show marked differences in ASV composition among the different treatments (Fig. 2), which was further confirmed by a lack of significance (PERMANOVA: F_3,32_ = 1.227, R^2^ = 0.103,* p* = 0.205). Overall, the structure of the bacterial communities remained stable under different levels of TBT supplementation. This contrasts with previous reports that showed that TBT supplementation increased the diversity parameters and/or was associated with differences in composition of the gut-associated bacterial communities of marine aquaculture shrimp (*Litopenaeus vannamei*)^36,37^, freshwater fish (*Cyprinus carpio*)^18^, and other animal hosts^38^.

**Figure 3:**
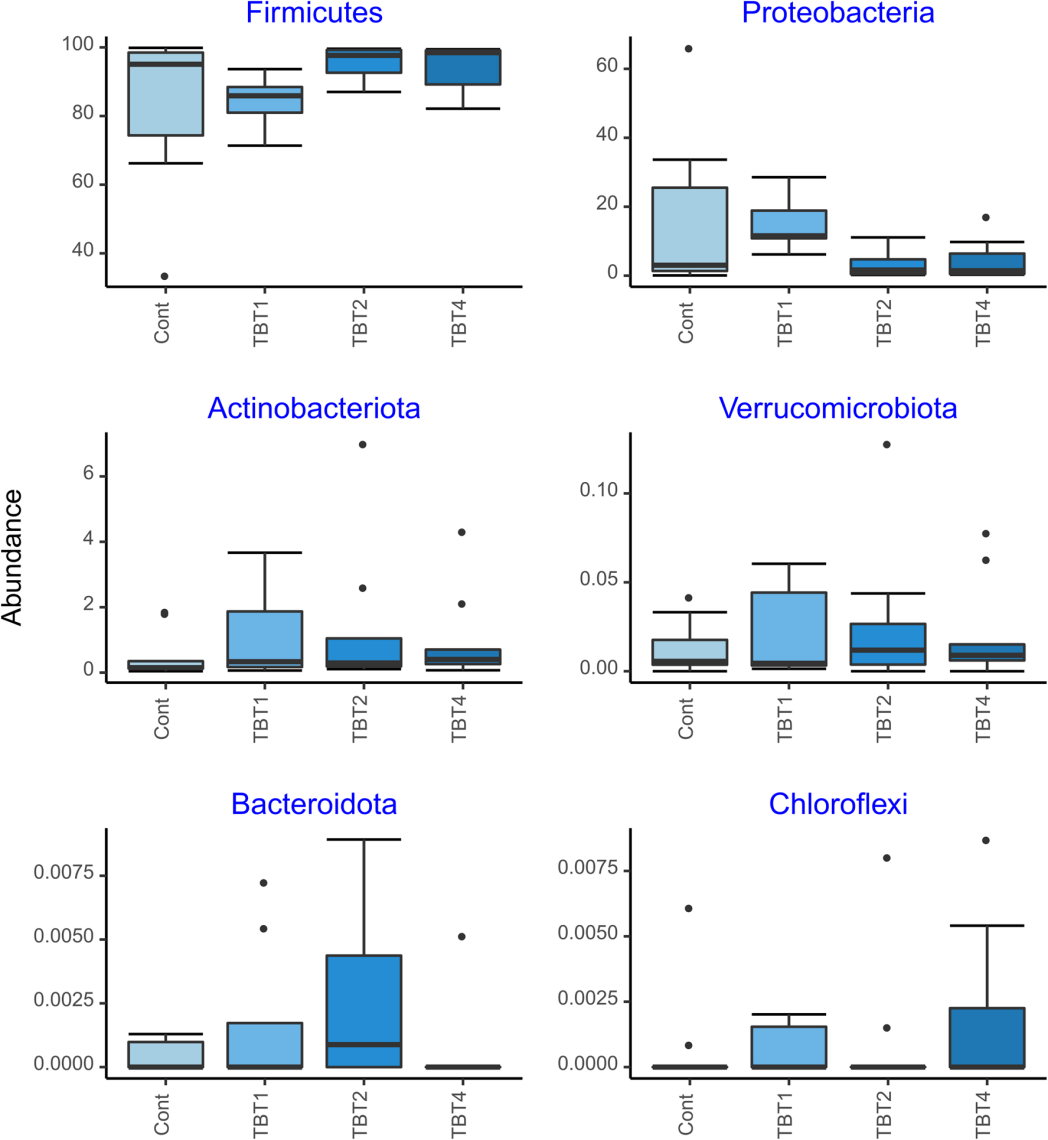
Boxplots of the relative abundances (%) of the six most abundant prokaryotic phyla found in the digesta-bacterial communities of fish fed diets with increasing levels of tributyrin (TBT). Symbols beyond the end of the whiskers are outliers. Cont: basal diet only (no TBT added); TBT1: basal diet with 0.1% TBT (m/m); TBT2: basal diet with 0.2% TBT (m/m); TBT4: basal diet with 0.4% TBT (m/m). In experimental diets, TBT was added to the basal diet at the expense of the excipient silica.

**Figure 4:**
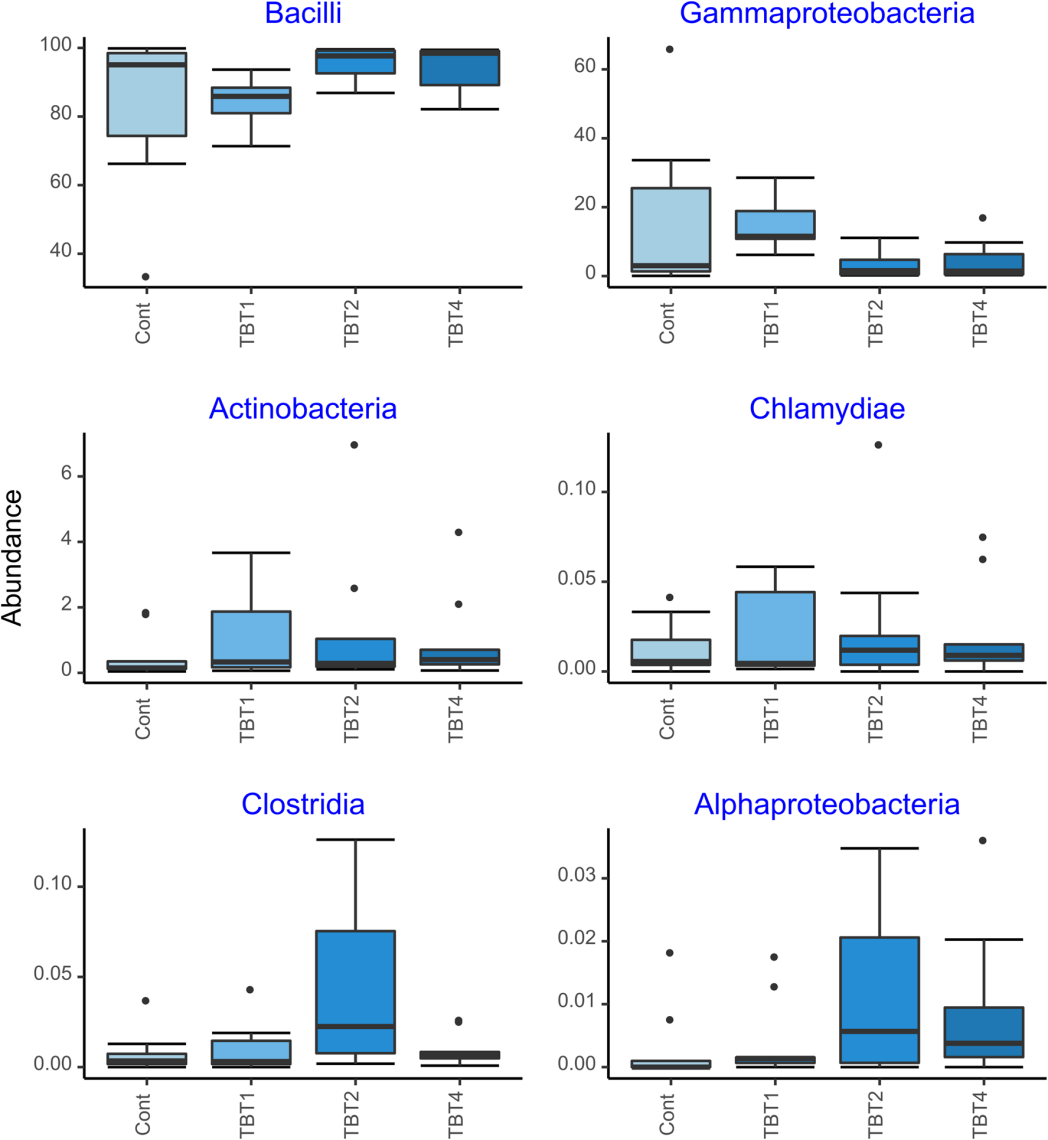
Boxplots of relative abundances (%) of the six most abundant prokaryotic classes found in the digesta-bacterial communities of fish fed diets with increasing levels of tributyrin (TBT). Symbols beyond the end of the whiskers are outliers. Cont: basal diet only (no TBT added); TBT1: basal diet with 0.1% TBT (m/m); TBT2: basal diet with 0.2% TBT (m/m); TBT4: basal diet with 0.4% TBT (m/m). In experimental diets, TBT was added to the basal diet at the expense of the excipient silica.

**Figure 5:**
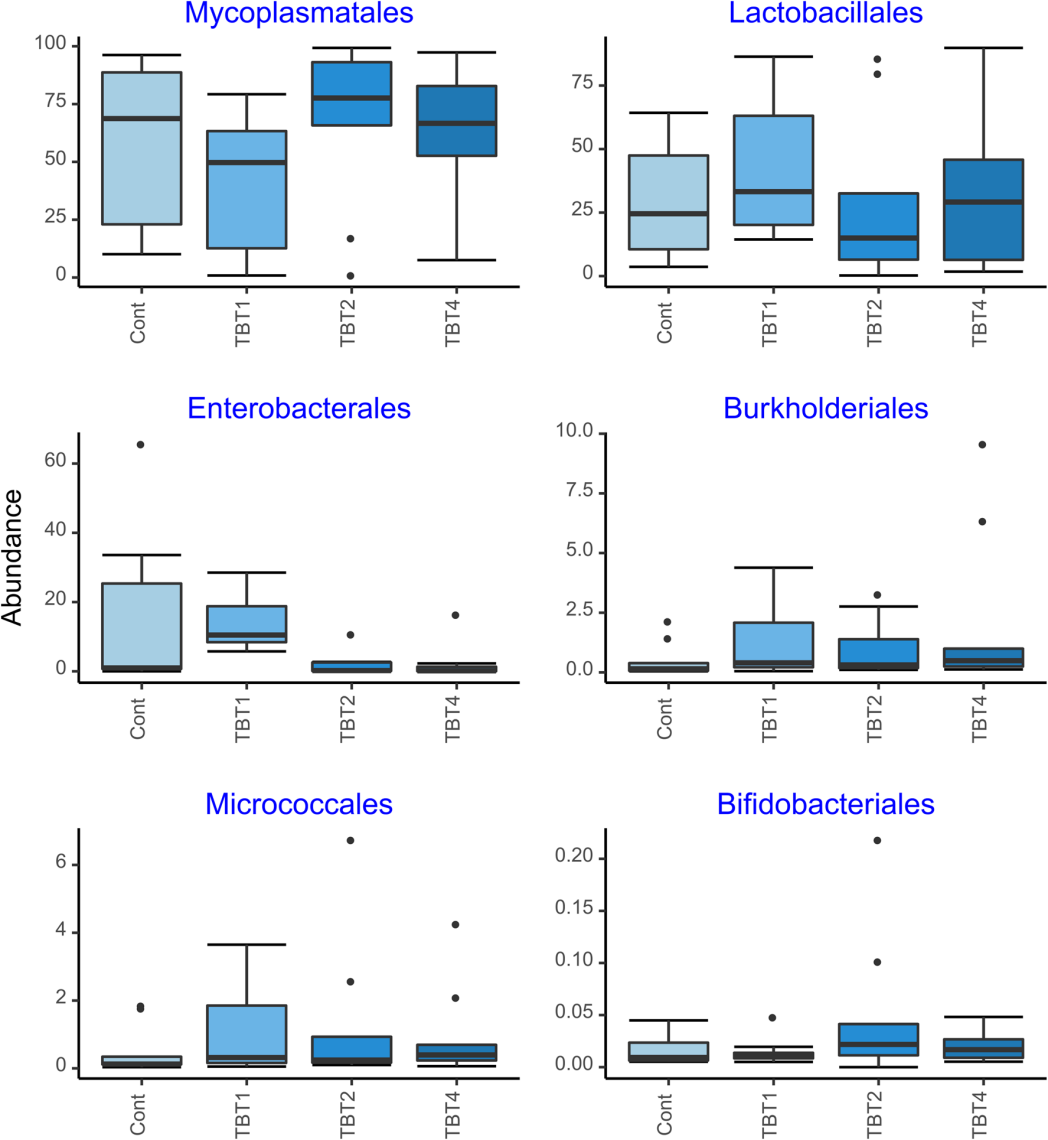
Boxplots of relative abundances (%) of the six most abundant prokaryotic orders found in the digesta-bacterial communities of fish fed diets with increasing levels of tributyrin (TBT). Symbols beyond the end of the whiskers are outliers. Cont: basal diet only (no TBT added); TBT1: basal diet with 0.1% TBT (m/m); TBT2: basal diet with 0.2% TBT (m/m); TBT4: basal diet with 0.4% TBT (m/m). In experimental diets, TBT was added to the basal diet at the expense of the excipient silica.

The compositional analysis of the bacterial communities detected a total of 14 phyla, with Firmicutes, Proteobacteria, Actinobacteriota, Verrucomicrobiota, Bacteroidota and Chloroflexi being the most abundant (Fig. 3). These results are consistent with other studies of the rainbow trout gut microbiome. For example, Brown et al. (2019) showed that Tenericutes (recently reclassified within the Firmicutes phyla), Spirochaetes, Proteobacteria and Firmicutes were the most abundant phyla in the mid-gut of rainbow trout^39^. The six most abundant classes were Bacilli, Gammaproteobacteria, Actinobacteria, Chlamydiae, Clostridia, and Alphaproteobacteria. The relative abundances of these taxa were similar among treatments (Fig. 4). The six most abundant orders were Mycoplasmatales, Lactobacillales, Enterobacterales, Burkholderiales, Micrococcales and Bifidobacteriales. Members of the Mycoplasmatales order have also been previously identified by Brown et al. (2019) as preponderant in the mid-gut of rainbow trout^39^. Of the most abundant orders, only the abundance of Enterobacteriales differed significantly among tributyrin supplementation treatments (GLM-ANOVA: F_3,32_ = 4.686, R^2^ = 7729.8, *p* = 0.008). Interestingly, the order Enterobacterales was significantly less abundant in the TBT2 and TBT4 treatments compared to the other treatments (EMMEANS: *P* < 0.05, Supplementary File S4, Fig. 5). Members of the Enterobacterales order are usually found in the gastrointestinal tracts of fish^39–41^. Overall these results are in line with a previous study demonstrating the ability of 1-monobutyrin supplementation to reduce the abundance of gammaproteobacterial populations^13^. However, in contrast to this study^13^, our findings indicate that the use of tributyrin as feed additive had no significant effect on the abundance of lactic acid bacteria (Lactobacilalles).

**Figure 6:**
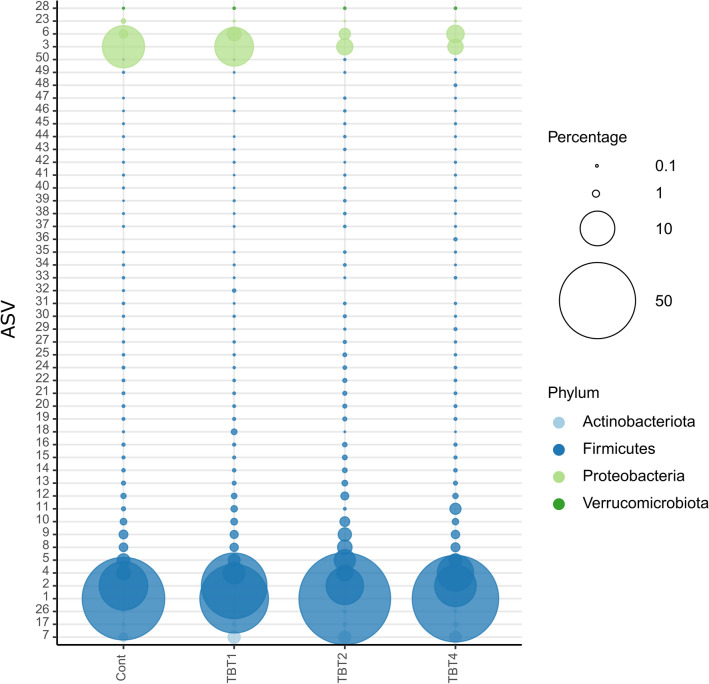
Relative abundances of the most abundant ASVs found in the digesta-bacterial communities of fish fed diets with increasing levels of tributyrin (TBT). ASVs were colour-coded according to their prokaryotic phylum. The circle size of the ASV is proportional to the mean percentage of sequences per treatment as indicated by the symbol legend in the upper right corner of the figure below the phylum assignment legend. Cont: basal diet only (no TBT added); TBT1: basal diet with 0.1% TBT (m/m); TBT2: basal diet with 0.2% TBT (m/m); TBT4: basal diet with 0.4% TBT (m/m). In experimental diets, TBT was added to the basal diet at the expense of the excipient silica.

Of the 50 most abundant ASVs, ASVs 1, 2, 3 and 4 were the most abundant in the fish gut (Fig. 6, Supplementary Fig. S1). We used the BLAST search tool to compare the sequences of these ASVs with those in the NCBI curated 16S gene sequence database (Supplementary File S3). Our results showed that ASVs 1 and 2 were abundant in all treatments. ASV-1 had low similarity to sequences of organisms identified as belonging to the *Malacoplasma* genus (Supplementary File S3). The genus *Malacoplasma* consists of certain strains previously classified to the genus *Mycoplasma*^42^. Meanwhile, ASV-2 was similar to sequences identified as belonging to the genus *Lactococcus*^43^, including *L. cremoris* subsp. *tructae,* which was originally isolated from the intestinal mucus of brown trout, and *L. lactis*^44^ (Supplementary File S3). *L. cremoris* strains have been shown in silico to produce substances that are antagonist to the common trout pathogen *L. garvieae* and both, *L. cremoris* and *L. lactis* have been proposed as candidate probiotics in freshwater aquaculture^45,46^.

Tributyrin supplementation exerted a significant influence on the abundance of ASV-3 (GLM-ANOVA: F_3,32_ = 4.657, R^2^ = 7781, *p* = 0.008) and ASV-4 (GLM-ANOVA: F_3,32_ = 3.845, R^2^ = 6328.7, *p* = 0.019). Diets with the highest TBT concentrations (TBT2 and TBT4) had significantly lower relative abundances of ASV-3 (EMMEANS: *p* < 0.05, Supplementary Fig. S1 and Supplementary File S4). ASV-3 was similar to different species of the Enterobacteriaceae family including *Kluyvera intermedia* and *Klebsiella pneumoniae* (Supplementary File S3). *Kluyvera intermedia* has been previously observed in association with the intestinal contents and mucus of rainbow trout^47,48^ suggesting that it could be part of the natural fish-associated microbial community. In humans, members of the *Kluyvera* genus colonize the gastrointestinal tract but are also capable of causing a series of infections^49^. *Klebsiella pneumoniae* has been previously associated with disease outbreaks in rainbow trout^50^ and rohu carp (*Labeo rohita*) in aquaculture systems^51^. This bacteria is an opportunistic pathogen that causes infections in fish with compromised immunities^52^. Within the aquaculture sector it is known that SCFA can reduce bacterial pathogen loads in fish by reducing the pH in the stomach and small intestine^53^. In a different context using mouse models, high levels of SCFA have been found to prevent the expansion of Enterobacteriaceae, including clinical isolates of *K. pneumoniae*^54,55^. In the specific case of *K. pneumoniae*, SCFAs led to intracellular acidification thus inhibiting its growth^55^. In parallel to the lower abundance of ASV-3 in TBT2 and TBT4, we also observed a significant increase in the relative abundance of ASV-4 in TBT4 in comparison to CONT and TBT2 (EMMEANS: *p* < 0.05, Supplementary Fig. S1 and Supplementary File S4). This ASV was similar to different species of the *Weissella* genus, including *W. hellenica,* and was previously isolated from flounder intestines and found to be a potential probiotic with antimicrobial activity against fish pathogens^56^.

In addition to the taxonomic analysis, we also predicted the metagenomic content of each sample. The proportion of sequences not used to predict the metagenomic content varied from 0.39 to 0.68. We specifically focused our analysis on KEGG categories relevant to fish health (e.g., biosynthesis of secondary metabolites, biosynthesis of antibiotics, biofilm formation). Several predicted pathways associated with pathogen colonization were depleted in TBT2 and TBT4 when compared to the control and TBT1 (Supplementary Fig. S3). For example, the relative gene counts of KEGG pathways related to biofilm formation in *Pseudomonas aeruginosa* (biofilm.pa) and *Escherichia coli* (biofilm.ec), and to the legionellosis disease were affected by tributyrin supplementation (GLM-ANOVA: *p* < 0.05; Supplementary Fig. S2). Post-hoc multiple comparison analysis found that gene counts for the biofilm.pa pathway were less abundant in TBT4 than in TBT2 (EMMEANS: *p* = 0.048; Supplementary File S4) and those for the legionellosis disease were less abundant in TBT2 than in the highest dosages of tributyrin (TBT2 and TBT4; EMMEANS: *p* < 0.05; Supplementary File S4). For biofilm.ec no significant differences were found between diets in the pairwise comparison (EMMEANS: *p* > 0.05). Bacterial biofilm formation is an important factor during pathogenic colonization. Communities of microorganisms in biofilms are less vulnerable to host immune responses than free-living cells and more resistant to antibiotics. Biofilm formation can, thus, be considered a virulence factor since it contributes to the ability to cause infection^57,58^. The legionellosis disease is not related to aquaculture settings. The presence of genes related to legionellosis pathway in the predicted functional profiles may be due to the presence of homologous genes from other proteobacterial ASVs. On the other hand, the increased level of TBT in fish diet (TBT2 and TBT4) was not associated with significant differences in gene count abundances of KEGG pathways related to antibiotic and secondary metabolite production (GLM-ANOVA: *p* > 0.05; Supplementary Fig. S3), although the mean abundance of these pathways tended to be higher in fish fed diets with the highest dosages of tributyrin (TBT2 and TBT4). This may play an important role in turning the gut environment more hostile to pathogen invasion and proliferation^59^.

Digestive enzyme activities in the gastrointestinal tract (GI) of the rainbow trout (24 h postprandial) are presented in Supplementary Fig. S4. Overall, our results showed that TBT had no significant effect on the activities of enzymes involved in the digestion of dietary carbohydrates (α-amylase), lipids (lipase), and proteins (trypsin and chymotrypsin; ANOVA: *p* > 0.05). The dietary supplementation of TBT has been previously shown to increase the activities of proteolytic enzymes in omnivorous marine fish and shrimp^36,37,60,61^. However, rainbow trout is highly carnivorous^62^ and, in other salmonids with similar dietary requirements (like Atlantic salmon *Salmo salar*), dietary supplementation with triglycerides only increased the activities of proteolytic enzymes with concentrations greater than those used in this study (> 0.4%)^63,64^. The effect of TBT supplementation on fish amylase and lipase activities is unclear, with some studies reporting significant increases^37,61^ and others no significant effect^36,60^. In relation to growth, no significant differences were found in the FBW (*p* = 0.765) among treatments.

Seventeen metabolites were identified in chyme samples by ^1^H NMR and are listed in the Supplementary Table S2. Regarding the PCA results of the metabolite profiles, all tested models showed that the groups were completely or almost completely superimposed (Supplementary Fig. S5). The univariate analysis (Kruskal–Wallis test) revealed that only taurine varied significantly among experimental groups (*p* = 0.0401). Taurine has been considered an indispensable nutrient for several carnivorous fish species when fed diets with high plant-meal contents^65^. However, no significant differences were identified by the post-hoc test (Dunn’s multiple comparison test: *p* < 0.05) on the pairwise comparisons (Supplementary Table S2). These results are generally in line with the analysis of digestive enzymes; the metabolomics analysis indicated that the final metabolite profiles of the aqueous fraction of the chyme were similar among dietary treatments.

## Conclusion

Overall, our results indicated that TBT did not significantly affect fish-gut bacterial community composition, digestive enzyme activities or digesta metabolite profiles when compared with non-supplemented controls. However, our results indicated that high TBT concentrations (TBT2 and TBT4) appeared to selectively inhibit members of the Enterobacterales order. In particular, higher levels of TBT were associated with a strongly reduced relative abundance of an Enterobacteriaceae ASV (ASV-3) which was closely related to the opportunistic pathogen *K. pneumoniae*. In addition to this, the predicted metagenomic analysis revealed that high levels of TBT were associated with lower gene count abundances in pathways related to pathogenesis. Altogether, our results highlight the potential suitability of TBT as a supplement in aquafeeds and encourage further studies to investigate the potential of this triglyceride to promote pathogen suppression in fish gut environments.

### Supplementary Information

Supplementary Information 1.

Supplementary Information 2.

Supplementary Information 3.

Supplementary Information 4.

Supplementary Legends.

## Data Availability

Sequences used in this study were uploaded to the NCBI ShortRead Archive (BioProject PRJNA909616; Biosamples SAMN32092074—SAMN32092109). The raw NMR spectra used in the current study have been uploaded to the Zenodo repository (https://zenodo.org) with the reference doi: 10.5281/zenodo.6671278.
