# Supplementary material for: Study of the influence of tributyrin-supplemented diets on the gut bacterial communities of rainbow trout (Oncorhynchus mykiss)
Source: Sci Rep. 2024 Mar 7;14:5645. doi: 10.1038/s41598-024-55660-y (PMC10920674; doi:10.1038/s41598-024-55660-y)
Supplement: Supplementary file 1 — Supplementary Information 1. [file 41598_2024_55660_MOESM1_ESM.docx]

>0a3cf58d4ca062c13d42c9db4ebcbc53

gacagaggatgcaagcgttatccggaatgattgggcgtaaagcgtctgtaggtggctttttaagttcgccgtcaaatcccagggctcaaccctggacaggcggtggaaactaccaagctggagtacggtaggggcagagggaatttccggtggagcggtgaaatgcgtagagatcggaaagaacaccaacggcgaaagcactctgctgggccgacactgacactgagagacgaaagctaggggagcgaatggg

>c0d5395792eadbf5f62e8ffb14fa0262

gacggggggggcaagtgttcttcggaatgactgggcgtaaagggcacgtaggcggtgaatcgggttgaaagtgaaagtcgccaaaaagtggcggaatgctctcgaaaccaattcacttgagtgagacagaggagagtggaatttcgtgtgtaggggtgaaatccgtagatctacgaaggaacgccaaaagcgaaggcagctctctgggtccctaccgacgctggggtgcgaaagcatggggagcgaacagg

>c0993401115369638c7bec4bec5981d7

tacgtagaagactagtgttaatcatctttattaggtttaaagggtacctagacggtaaattaaactctaaatgagtacttgtttactagagttttatgtaaggaggaagaatttctggagtagtgatttaatatgaataatctcagagagactggtaacggcgaaggcatccttctatgtaaaaactgacgttgagggacgaaggcttgggtagcgagaagg

>7911816f5e81f650f769aba0d5c708cb

gacagaggatgcaagcgttatccggaatgattgggcgtaaagcgtctgtaggtggcttttcaagtccgccgtcaaatcccagggctcaaccctggacaggcggtggaaactaccaagctggagtacggtaggggcagagggaatttccggtggagcggtgaaatgcattgagatcggaaagaacaccaacggcgaaagcactctgctgggccgacactgacactgagagacgaaagctaggggagcaaatggg

>22f1fa0bdcc19746dee080bcc12a1840

gacggggggggcaagtgttcttcggaatgactgggcgtaaagggcacgtaggcggtgaatcgggttgaaagtgaaagtcgccaaaaactggtggaatgctctcgaaaccaattcacttgagtgagacagaggagagtggaatttcgtgtgtaggggtgaaatccgcagatctacgaaggaacgccaaaagcgaaggcagctctctgggtccctaccgacgctggagtgcgaaagcatggggagcgaacggg

>e679d286d455a87f211f907f247f0ce6

acggggggggcaagtgttcttcggaatgactgggcgtaaagggcacgtaggcggtgaatcgggttgaaagtgaaagtcgccaaaaagtggcggaatgctctcgaaaccaattcacttgagtgagacagaggagagtggaatttcgtgtgtaggggtgaaatccgtagatctacgaaggaacgccaaaagcgaaggcagctctctgggtccctaccgacgctggggtgcgaaagcatggggagcgaacagg

>ba48cb83459c7305cd4f8f9c50147fbd

gacagaggatgcaagcgttatccggaatgattgggcgtaaagcgtctgtaggtggctttttaagttcgctgtcaaataccagggctcaaccctggacaggtggtgaaaactactaagctagagtacggtaggggcagagggaatttccggtggagcgatgaaatgcgtagagatcggaaggaacaccaacggcgaaagcactctgctgggccgacactgacactgagagacgaaagctaggggagcgaatggg

>bf6012867f37a53e6099f1ada6791477

tacataggatgcaagcgttatccggaatgattgggcgtaaagcgtctgtaggtggctttttaagtccgccgtcaaatcccagggctcaaccctggacaggcggtggaaactaccaagctggagtacggtaggggcagagggaatttccggtggagcggtgaaatgcgcagagatcggaaagaacaccaacggcgaaagcactctgctgggccgacactgacactgagagacgaaagctaggggagcgaatggg

>d967ac7635542ce8061e9bc0f70e2823

tacgtagaagacaagtgttattcatctttaacaggtttaaagggtacctagacggaaaatcaagccatagtagggactagttttctagagttttatgtgtgaagatcgaattacctgaagagcaataaaatgcattgacacagggaagacgggtagcagcgaaggcaatcttctatgtagaaactgacgttgagggacgaagccttggggagcgagaagg

>19bd3046e57e0f2acbf22eea5a60666b

gacggggggggcaagtgttcttcggaatgactgggcgtaaagggcacgtaggcggtgaatcgggttgaaagtggaagtcgccaaaaagtggcggaatgctctcgaaaccaattcacttgagtgagacagaggagagtggaattccatgtgtagcggtgaaatgcgtagatatatggaggaacaccggtggcgaaagcggctctctggcctgtaactgacactgaggctcgaaagcgtggggagcaaacagg

>d990d01464aabbda108c9ed7372949df

gacaagggagacgagtgttattcatctttaacaggtatatagggtacctagacggtgtgcaatggcttacataagtacctggtacacttgagtttgatatgtgagaggaatatgtcggaattgttggaggaaagatgaaattttttgataccaataggaccggtaacggcgaaggcaaacctctatgtacatgtacttgctatttctctagtataaagaggaaaagagcaatgtactataactgacgttgagggacgaaggctcagatagtgaagagg

>cf5d5fd0a1c89359ac49b2d598fc8203

gacagaggatgtaagcgttatccggaatgattgggcgtaaagcgtctgtaggtggctttttaagttcgccgtcaaatcccagggctcaaccctggacaggcggtggaaactaccaagctggagtacggtaggggcagagggaatttccggtggagcggtgaaatgcgtagatatatggaagaacaccagaggcgaaggcgaaaacttaggctataactgacgcttaggctcgaaagtgtggggagcaaatagg

>79577c5412396fde41d64fd4bd9d31b3

gacagaggatgcaagcgttatccggaatgattgggcgtaaagcgtctgtagatggctttttaagttcgccgtcaaatcccagggctcaaccctggacaggcggtggaaactaccaagctggagtacggtaggggcagagggaatttccggtggagcggtgaaatgcgtagatatatggaagaacaccagtggcgaaggcggctttctggactgtaactgacgttgaggctcgaaagtgtgggtagcaaacagg

>863082d3d9a68dbfe011b0ef04282b58

tacagaggatgcaagcgttatccggaatcactgggcataaagcgtctgtaggttgcttgccaagtctgctgttaaagatcagggcttaaccctgggaaagcagtggaaactagtaggcttgagtgtggtagaggtagagggaattcctggtgtagcggtgaaatgcgtagatattaggaagaacaccaatggcgaaagcactctactgggccacaactgacactgagagacgacagctaggggagcaaatggg

>c638f5b29780fa08a711eb24c5f0c896

gacagaggatgcaagcgttatctggaatgattgggcgtaaagtgtctgtaggtggcttttcaagtgcgccgtcaaatcccagggctcaatccgggacaggcggtggaaactaccaagctggagtacggtaggggtagagggaatttctggtagagcggtgaaatgcattgagatcggaaagaacaccaacggcgaaagcactctgctgggcctacactgacactgagagatgaaagctatgggagcaaatggg

>02ec9e816407b031507396c81c182019

gacggaggatgcaagtgttatccggaatcactgggcgtaaagcgtctgtaggtggtttaataagtcaactgttaaatcttgaggcttaacttcaaaatcgcagtcgaaactattagactagagtatagtaggggtaaagggaatttccagtggagcggtgaaatgcgtagagattggaaagaacaccgatggcgaaggcactttactgggctattactaacactcagagacgaaagctagggtagcaaatggg

>e328998b44f14f84d6205d33c9c420c0

tacaagtcagactagtgttattcatctttattaggtttaaagggtacctagacagtctatctcgcctgcttattatagcgaaggaacagatttatgactagagttttatgggagaggtaaatattaggaagagcagtgtagagataaaattttttaatactcttaggacgtataacggcgaaggcaaacctctatgtaaaaactgacgttaagggacgaaggcttgggtagcgaaaagg

>869f73a09755086b01c2013fd4fec375

gacggggggggcaggtgttcttcggaatgactgggcgtaaagggcacgtaggcggtgaatcgggttgaaagtgaaagtcgccaaaaagtggcggaatgctctcgaaaccaattcacttgagtgagacagaggagagtggaattccatgtgtagcggtgaaatgcgtagatatatggaggaacaccggtggcgaaagcggctctctggcctgtaactgacactgaggctcgaaagcgtggggagcaaacagg

>459442543782ddc03bb001438b297dba

gacggaggatgcaagtgttatccggaatcactgggcgtaaagcgtctgtaggtggtttgataagtcaactgttaaatcttgaagctcaacttcaaaatcgcagtcgaaactatcaggctagagtatagtaggggtaaagggaatttccagtggagcggtgaaatgcgtagatattggaaagaacaccgatggcgaaggcactttactgggctattactgacactcagagacgaaagctagggtagcaaatggg
