# Supplementary material for: Study of the influence of tributyrin-supplemented diets on the gut bacterial communities of rainbow trout (Oncorhynchus mykiss)
Source: Sci Rep. 2024 Mar 7;14:5645. doi: 10.1038/s41598-024-55660-y (PMC10920674; doi:10.1038/s41598-024-55660-y)
Supplement: Supplementary file 2 — Supplementary Information 2. [file 41598_2024_55660_MOESM2_ESM.docx]

Supporting information

Table S1 - Ingredients and nutrient content of the experimental diets. Cont: basal diet only (no TBT added); TBT1: basal diet with 0.1% TBT (m/m); TBT2: basal diet with 0.2% TBT (m/m); TBT4: basal diet with 0.4% TBT (m/m). TBT product contained 45% silica carrier and was supplied by Lucta S.A. (Spain). TBT product was added to Cont diet with the according adjustment for silica.

| **Ingredients, %** | **CONT** | **TBT1** | **TBT2** | **TBT4** |
| --- | --- | --- | --- | --- |
| Fishmeal Super Prime | 10.0 | 10.0 | 10.0 | 10.0 |
| Soy protein concentrate (Soycomil P) | 12.5 | 12.5 | 12.5 | 12.5 |
| Pea protein concentrate 80 (SP) | 12.5 | 12.5 | 12.5 | 12.5 |
| Wheat gluten | 13.0 | 13.0 | 13.0 | 13.0 |
| Corn gluten meal | 7.5 | 7.5 | 7.5 | 7.5 |
| Soybean meal 44 | 5.0 | 5.0 | 5.0 | 5.0 |
| Wheat meal | 9.35 | 9.35 | 9.35 | 9.35 |
| Wheat bran | 4.0 | 4.0 | 4.0 | 4.0 |
| Potato starch gelatinised | 6.35 | 6.35 | 6.35 | 6.35 |
| Vit & Min Premix PV01 | 1.0 | 1.0 | 1.0 | 1.0 |
| Vitamin E50 | 0.05 | 0.05 | 0.05 | 0.05 |
| Betaine HCl | 0.05 | 0.05 | 0.05 | 0.05 |
| Antioxidant powder (Verdilox) | 0.2 | 0.2 | 0.2 | 0.2 |
| MAP (Monoammonium phosphate) | 1.5 | 1.5 | 1.5 | 1.5 |
| L-Lysine HCl 99% | 0.5 | 0.5 | 0.5 | 0.5 |
| DL-Methionine | 0.2 | 0.2 | 0.2 | 0.2 |
| **Tributyrin product ^*^** | **-** | **0.1** | **0.2** | **0.4** |
| **Silica ^**^** | **0.4** | **0.3** | **0.2** | **0.0** |
| Fish oil | 3.0 | 3.0 | 3.0 | 3.0 |
| Rapeseed oil | 12.9 | 12.9 | 12.9 | 12.9 |
| **Total** | **100** | **100** | **100** | **100** |
|  |  |  |  |  |
| **Analyzed nutrient content** | **CONT** | **TBT1** | **TBT2** | **TBT4** |
| Crude protein, % feed | 44.0 | 44.0 | 44.0 | 44.0 |
| Crude fat, % feed | 18.0 | 18.0 | 18.0 | 18.0 |
| Fiber, % feed | 1.4 | 1.4 | 1.4 | 1.4 |
| Starch, % feed | 17.1 | 17.1 | 17.1 | 17.1 |
| Ash, % feed | 5.2 | 5.2 | 5.2 | 5.2 |
| Gross energy, MJ/kg feed | 21.8 | 21.8 | 21.8 | 21.8 |

* Consisting of 98% purity TBT (55%) and silica carrier (45%) supplied by Lucta S.A. (Spain).

** Same as found in Tributyrin product


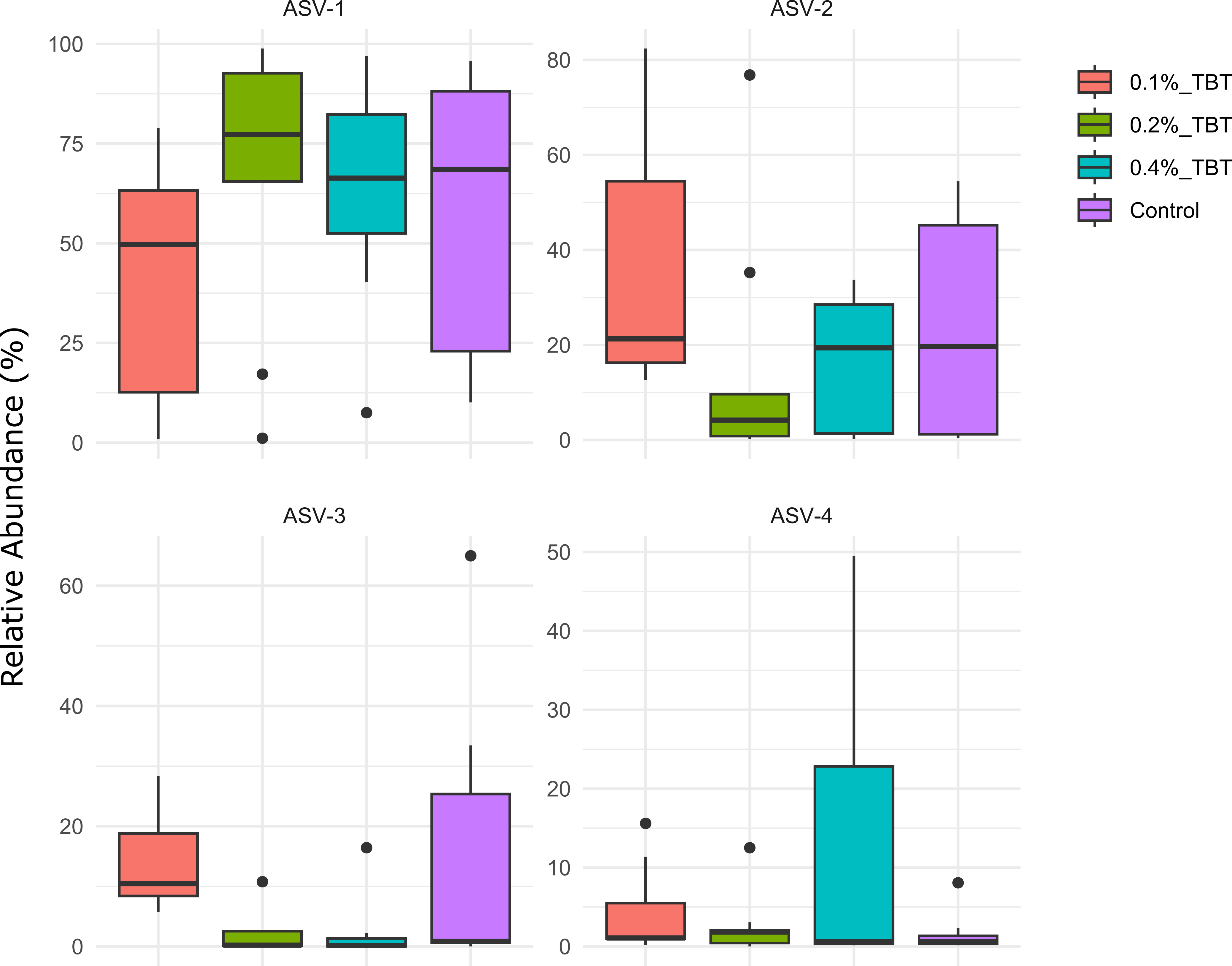


Figure S1: Boxplot of the relative abundance of the four most abundant ASV in the dataset. Control: basal diet only (no TBT added); 0.1%_TBT: basal diet with 0.1% TBT (m/m); 0.2%_TBT: basal diet with 0.2% TBT (m/m); 0.4%_TBT: basal diet with 0.4% TBT (m/m). In experimental diets TBT was added to basal diet at the expense of the excipient silica.

**

Figure S2: Boxplot of the variation in the relative gene count abundance for KEEG pathways: Quorum sensing (Quorum); Biofilm formation - Pseudomonas aeruginosa (Biofilm.Pa), Biofilm formation - Escherichia coli (Biofilm.Ec), Biofilm formation - Vibrio cholerae (Biofilm.Vc), Salmonella infection (Salmonella) and Legionellosis. Data beyond the end of the whiskers are "outlying" points and are plotted individually. Cont: basal diet only (no TBT added); TBT1: basal diet with 0.1% TBT (m/m); TBT2: basal diet with 0.2% TBT (m/m); TBT4: basal diet with 0.4% TBT (m/m). In experimental diets TBT was added to basal diet at the expense of the excipient silica.

Figure S3: Boxplot of the variation in the relative gene count abundance for KEEG pathways: Biosynthesis of secondary metabolites (Metabolites), Biosynthesis of antibiotics (Antibiotics), Carbon metabolism (Carbon), Nitrogen metabolism (Nitrogen), Sulfur metabolism (Sulfur) and Two-component system (Two.component). Cont: basal diet only (no TBT added); TBT1: basal diet with 0.1% TBT (m/m); TBT2: basal diet with 0.2% TBT (m/m); TBT4: basal diet with 0.4% TBT (m/m). In experimental diets TBT was added to basal diet at the expense of the excipient silica. Data beyond the end of the whiskers are "outlying" points and are plotted individually.

Figure S4: Digestive enzyme activities of rainbow trout fed diets with increasing levels of tributyrin (TBT) inclusion. Activities of (A) amylase, (B) lipase, (C) trypsin and (D) chymotrypsin were quantified in the whole intestine of the fish 24 h post-feeding. Cont: basal diet only (no TBT added); TBT1: basal diet with 0.1% TBT (m/m); TBT2: basal diet with 0.2% TBT (m/m); TBT4: basal diet with 0.4% TBT (m/m). In experimental diets TBT was added to basal diet at the expense of the excipient silica. Boxes represent the upper 75th and lower 25th percentiles. Within each box the black represents the median value (n=9). The whisker bars indicate the 90th and 10th percentiles.

Table S2. Metabolites identified in the digesta samples by ^1^H NMR and comparison of their relative concentration. Comparison performed applying the Kruskal-Wallis test and the Dunn’s multiple comparison post-test in groups with significant variations. Cont: basal control diet, TBT1: diet supplemented with tributyrin at 0.1%, TBT2: diet supplemented with tributyrin at 0.2%, TBT4: diet supplemented with tributyrin at 0.4%, ns: non-significant, *: p < 0.05.

| Metabolite | Kruskal-Wallis  (p-value) | Dunn's Multiple Comparison Test | | | |
| --- | --- | --- | --- | --- | --- |
|  |  | Cont | TBT1 | TBT2 | TBT4 |
| Valine | ns (0.8859) | - | - | - | - |
| Leucine | ns (0.824) | - | - | - | - |
| Isoleucine | ns (0.2907) | - | - | - | - |
| Isobutyrate | ns (0.7265) | - | - | - | - |
| Threonine/Lactate | ns (0.8349) | - | - | - | - |
| Alanine | ns (0.9514) | - | - | - | - |
| Acetate | ns (0.4433) | - | - | - | - |
| Acetylcholine | ns (0.8732) | - | - | - | - |
| Acetoacetate | ns (0.3873) | - | - | - | - |
| TMAO | ns (0.2266) | - | - | - | - |
| Betaine | ns (0.9708) | - | - | - | - |
| Taurine | * (0.0401) | ns | ns | ns | ns |
| Glycerol | ns (0.1524) | - | - | - | - |
| Maltose | ns (0.2958) | - | - | - | - |
| Tyrosine | ns (0.1729) | - | - | - | - |
| Phenylalanine | ns (0.5811) | - | - | - | - |
| Formate | ns (0.7679) | - | - | - | - |


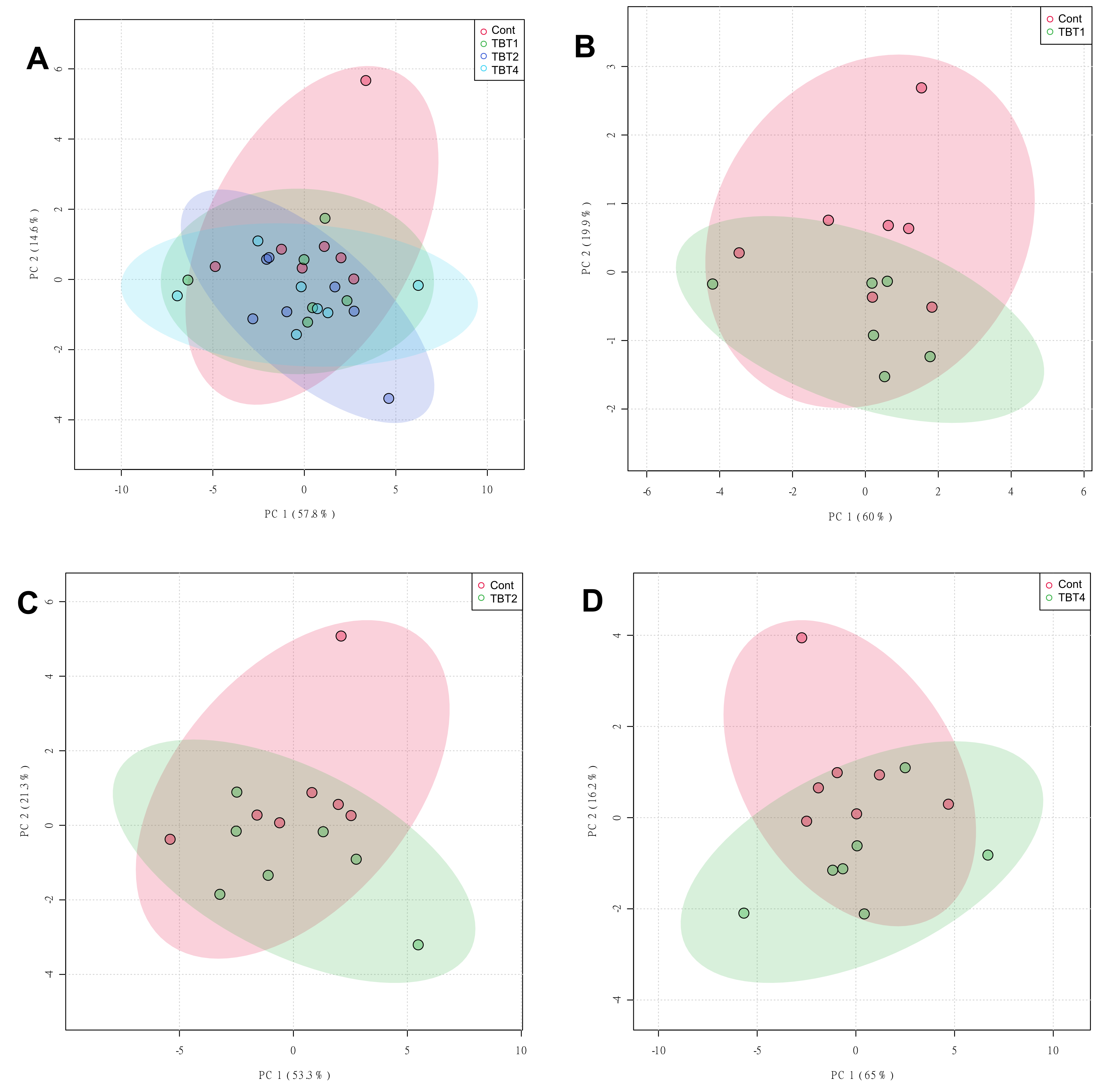


Figure S5. Principal Component Analysis (PCA) computed with the relative concentration values of each metabolite of: A) all experimental groups 24h, B) Cont and TBT1 groups, C) Cont and TBT2 groups, D) Cont and TBT4 groups. All ellipses in the scores plots were drawn at the 95% confidence level. Four spectra from group Cont, one from group TBT1, two from group TBT2 and two from group TBT1 were not considered for analysis due to its poor quality. Cont: basal control diet (n = 5), TBT1: diet supplemented with tributyrin at 0.1% (n = 8), TBT2: diet supplemented with tributyrin at 0.2% (n = 7), TBT4: diet supplemented with tributyrin at 0.4% (n = 7).
