# Supplementary material for: Study of the influence of tributyrin-supplemented diets on the gut bacterial communities of rainbow trout (Oncorhynchus mykiss)
Source: Sci Rep. 2024 Mar 7;14:5645. doi: 10.1038/s41598-024-55660-y (PMC10920674; doi:10.1038/s41598-024-55660-y)
Supplement: Supplementary file 5 — Supplementary Legends. [file 41598_2024_55660_MOESM5_ESM.docx]

Supplementary Files:

Supplementary File S1: List of deleted ASVs prior to statistical analysis.

Supplementary File S2: File containing Supplementary Table S1 and Supplementary Figures S1-S4

Supplementary File S3: Blast analysis of ASV1, ASV2, ASV3 and ASV4. The top 100 results are showed

Supplementary File S4: Results from the multiple comparisons using emmeans function for diversity indices and relative abundances of most abundant phyla, classes, orders and ASVs.
